# In silico evolution of diauxic growth

**DOI:** 10.1186/s12862-015-0492-0

**Published:** 2015-09-29

**Authors:** Dominique F. Chu

**Affiliations:** School of Computing, University of Kent, Canterbury, CT2 7NF UK

**Keywords:** Diauxic growth, Glucose effect, Simulated evolution

## Abstract

**Background:**

The glucose effect is a well known phenomenon whereby cells, when presented with two different nutrients, show a diauxic growth pattern, i.e. an episode of exponential growth followed by a lag phase of reduced growth followed by a second phase of exponential growth. Diauxic growth is usually thought of as a an adaptation to maximise biomass production in an environment offering two or more carbon sources. While diauxic growth has been studied widely both experimentally and theoretically, the hypothesis that diauxic growth is a strategy to increase overall growth has remained an unconfirmed conjecture.

**Methods:**

Here, we present a minimal mathematical model of a bacterial nutrient uptake system and metabolism. We subject this model to artificial evolution to test under which conditions diauxic growth evolves.

**Results:**

As a result, we find that, indeed, sequential uptake of nutrients emerges if there is competition for nutrients and the metabolism/uptake system is capacity limited.

**Discussion:**

However, we also find that diauxic growth is a secondary effect of this system and that the speed-up of nutrient uptake is a much larger effect. Notably, this speed-up of nutrient uptake coincides with an overall reduction of efficiency.

**Conclusions:**

Our two main conclusions are: (*i*) Cells competing for the same nutrients evolve rapid but inefficient growth dynamics. (*ii*) In the deterministic models we use here no substantial lag-phase evolves. This suggests that the lag-phase is a consequence of stochastic gene expression.

## Background

Diauxic growth is perhaps one of the best known biological phenomena. Its discovery goes back to Monod [1] who found that on a mix of glucose and lactose *E.coli* first grows exponentially on the preferred nutrient (i.e. glucose), then stops growing and then resumes, somewhat slower, exponential growth fuelled by the less preferred nutrient. The phase in-between the exponential growth episodes is called the *lag-phase*. Diauxic growth and the network that controls it has been subject to intense experimental [2–8] and theoretical [5, 9–11] investigation. Most of this work has focussed on understanding the detailed mechanisms controlling diauxie.

There are two main mechanisms responsible for the two phase growth in bacteria, both of which depend on the *phototransferase* (PTS) system [12]. Firstly, regulation of metabolic genes via global transcription regulators, especially cAMP. Secondly, direct uptake mediated inducer exclusion. In *E.coli* the levels of dephospho EIIA ^Glc^ increase during glucose uptake. EIIA ^Glc^ inactivates the uptake of the secondary sugars (i.e. lactose) and in this way prevents the induction of the relevant uptake system.

Given the complex regulatory interactions that implement diauxic growth one is led to assume that it has some adaptive significance, i.e. it is not simply an evolutionary frozen accident. It is commonly conjectured that diauxic growth enables cells “to increase their fitness by optimizing growth rates in natural environments providing complex mixtures of nutrients” [2]. Yet precisely under which condition two phase growth is adaptive is usually left unspecified. Similarly, it is rarely discussed in detail why and when the lag-phase is adaptive: In Monod’s original experiments it lasts for about 20 minutes which is of the order of magnitude of a typical generation time in exponentially growing *E.coli*. Halting growth for such a long period of time comes with a substantial fitness penalty. It is not clear how this growth cost can be counter-balanced by other effects.

One hypothesis is that the lag-phase is simply an unavoidable consequence of the switch between nutrient sources. Yet, this does not explain the lag-phase itself. For one, one could image that cells start to switch to the second nutrient before the first nutrient is completely exhausted. There would be a short period of simultaneous uptake of nutrient and an immediate uptake of the second nutrient with no cessation of growth. Furthermore, there is also emerging new experimental evidence [8] that the lag-phase is under evolutionary control and is linked to stochastic effects of gene expression. If found confirmed, this would suggest that the lag-phase is not explainable by deterministic population level models but perhaps requires explanatory approaches based on the metabolic cost of rapid state changes in stochastic computers [13].

Uptake and metabolism of nutrient is not free, but requires a specific uptake machinery to be produced and hence comes at an energetic cost. Uptake withdraws nutrient from general growth and cell division; the faster the uptake the higher the cost. This poses the question as to what the best allocation strategy might be: if the cell channelled all nutrients into growth, but used none for uptake, then it would starve in an ocean of nutrient. Similarly, if all nutrients were to be committed to uptake but none to growth, then the cell would not be able to grow either. This suggests the existence of a sweet-spot balancing uptake and growth. Indeed, for the case of the *lac* operon this optimum was shown to exist in artificial wet-lab evolution [14, 15]. In the case of non-constant environments, including environments with two nutrients, there is then no longer a single optimum but an optimal trajectory of states. The question then arises what the best way of switching between different states [16] depending on the statistical properties of the environment [17].

While stochastic effects most likely have a role to play for the evolution of diauxic growth, we argue here that it is not the main effect. Instead, resource allocation is a primary driver of diauxic growth: it is not possible to understand how much energy the cell should expend on stochastic regulation without understanding the overall principles of resource allocation; vice-versa, it *is* possible to establish how the cell should best allocate resource without knowing the specifics of the costs of stochastic gene regulation. Furthermore, the mathematics of stochastic gene regulation is difficult and simulations are computationally expensive despite recent progress in the field [18, 19].

In order to understand resource allocation in cells (and hence diauxic growth), we present here a model of nutrient uptake and metabolism that is based on the PTS system and as such captures the essential aspects of nutrient uptake in bacteria. We only specify the topology of the network (i.e. which proteins interact, which pathways exist) and use artificial evolution to obtain the parameters for the model. Each of the evolved parameter sets can be interpreted as a resource allocation strategy. Crucial for our conclusions is that the artificial evolution algorithm we use implements competition for shared resources. Such competition is essential for the understanding of diauxic growth.

We will find that diauxic growth, or more precisely sequential nutrient uptake does evolve but only when the cell is under competitive pressure. At the same time, competitive pressure is only a necessary condition for sequential uptake to evolve, not a sufficient one. Diauxic growth will only evolve when the capacity of the uptake system or metabolism is strictly limited, for example if there is limited space for porins on the cell surface. More importantly, our artificial evolution simulations show that sequential uptake is only a secondary adaptation to competition in a dual nutrient environment. The primary effect is a speed up of the nutrient uptake and metabolism of the cell. At least within our set-up, this speed-up is the main contributor to the fitness of the cell in a competitive environment.

Interestingly, the increase of the uptake speed over evolutionary time-scale leads to an overall decrease of the cell efficiency. The fast growth forces the cell from the optimal resource allocation. Altogether, competitive evolution therefore leads to less efficient cells. Yet, while wasting nutrients, these faster growing cells are more competitive than their more efficient variations.

## Results

### The basic model

**Fig. 1:**
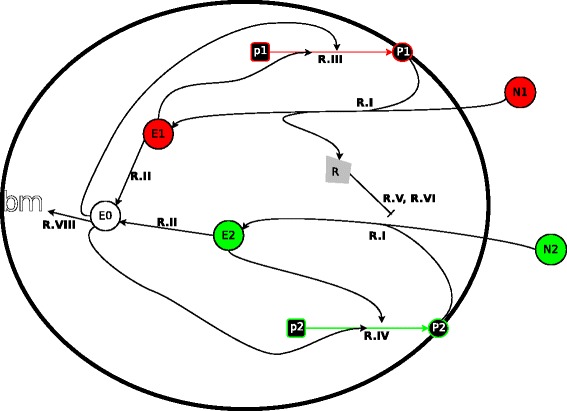
Graphical representation of the topology of the model. To maintain clarity of the diagram, the breakdown reactions R.VII are not represented. Moreover, the repression mechanism of reactions R.V and R.VI involving the formation of *B*_2_ from permease and repressor is only indicated by a repressor symbol

Throughout this contribution we will use a fixed network topology implementing a simplified model of inducer exclusion based on the PTS system (see reaction scheme Fig. 1 and Table 1). There are two nutrients on offer, one of which affords a higher growth to the cell. These external nutrients are converted into some internal cellular resource/energy (“ATP”) that can then itself be converted into biomass (and hence growth). Crucially, internal energy is also required for gene expression and hence for the generation of the uptake machinery. This entails that there is an inherent trade-off between the amount of nutrient invested in growth and the amount of nutrient used for the uptake machinery.

**Table 1:** The network topology formulated as a system of chemical equations. Note that *R*_2_=*∅* and is not represented in the actual model but merely introduced here for ease of notation. The factor *L* is defined in Eq. 1. The symbols *p*_*i*_ represent the gene from which the porins are transcribed. The factor $ \mathcal {H}:= {E_{0}^{2}}/(0.001^{2} + {E_{0}^{2}})$ denotes a Hill function; it prevents gene expression when there is no internal energy in the cell. In all experiments reported here $d_{\protect \{P_{i},E_{i}\protect \}}$ were set proportional to the growth rate, i.e. the reaction rate of R.VIII, while *d*_*R*_ is an evolvable parameter acting on a much faster time-scale

Substrate	Product	Reaction rate	Reaction number
*N*_*i*_	*E*_*i*_+*R*_*i*_	$ k_{N_{i}} \frac {N_{i}}{N_{i} + K_{N_{i}}} P_{i}\, bm $	R.I
*E*_*i*_	*E*_0_	$ k_{E_{i}} E_{i}$	R.II
*p*_1_+*E*_0_	*p*_1_+*P*_1_	$\left (\textrm {leak1} + k_{P_{1}} {\frac {E_{1}^{H}}{{E_{1}^{H}} + K_{P_{1}}^{H}}} E_{0}\right) L \, bm \, \mathcal {H} $	R.III
*p*_2_+*E*_0_	*p*_2_+*P*_2_	$\left (\textrm {leak2} + k_{P_{2}} {\frac {E_{2}^{H}}{{E_{2}^{H}} + K_{P_{2}}^{H}}} {\frac {K_{R}^{H}}{R^{H} + {K_{R}^{H}}}} E_{0}\right)L\, bm \, \mathcal {H}$	R.IV
*R*_1_+*P*_2_	*B*_2_	*k*_*b*_ *R*_1_ *P*_2_	R.V
*B*_2_	*R*_1_+*P*_2_	*k*_*ub*_ *B*_2_	R.VI
{*P*_*i*_,*E*_*i*_,*R*_1_}	*∅*	$d_{\{P_{i},E_{i}, R_{1}\} }$	R.VII
*E*_0_	*b* *m*	$ g\, bm \frac {E_{0}}{E_{0} + {K_{g}}}$	R.VIII

The network topology of the model allows for repression of uptake of the inferior model by a repressor that directly binds to the specific porins thus disabling them. The amount of repressor produced is proportional to the uptake of the primary nutrient. Furthermore, the repressor also directly represses expression of the porin for the inferior nutrient. These mechanisms reflect in a simplified form the dual repression in the PTS system of *E.coli*, described above.

The network, as expressed by equivalent chemical reactions is described in Table 1; a full set of differential equations including the relevant Maple 17 files can be obtained on request from the author. The reaction system assumes two sources of nutrients *N*_1_ and *N*_2_, where we assume that *N*_1_ is the more valuable one. Uptake of these sources of nutrients requires specific porins, namely *P*_1_ and *P*_2_ respectively (R.I). Once taken up into the cell the nutrient becomes an internal source of energy (*E*_1_ and *E*_2_) which can be converted into actual energy; this happens in reaction R.II. We denote the internal energy by *E*_0_. Within the cell *E*_0_ is converted either into porins (*P*_1_, *P*_2_) (in reactions R.III and R.IV respectively) or into biomass (*bm*) (in R.VIII).

The expression of porins in R.III and R.IV is activated by the presence of the respective nutrients inside the cell (*E*_1_ and *E*_2_). As such nutrient uptake is auto-activating which is a commonly observed regulatory motif in bacterial metabolic genes [20]. We assume that the activation function is of a Hill-type [21] with the fixed Hill exponent *H*=2, the evolvable Hill constant *K*, and maximal expression rates $k_{P_{1}}$ and $k_{P_{2}}$ for *P*_1_ and *P*_2_ respectively. The Hill constant determines how much internal nutrient is required to switch the system on and the maximal uptake rate determines how fast external nutrient is taken up when the system is switched on. In addition to a regulated activation of the uptake system, we also allow the cell to evolve a constitutive leak expression of the expression apparatus, i.e. parameters *leak*1 and *leak*2. Growth is represented by reaction R.VIII where internal energy is converted into biomass *bm*.

A component of central importance is the regulator *R*. Uptake of *N*_1_ in R.I coincides with the dephosphorylation of *R*^*p*^ thus producing the regulator *R*. The dephosphorylated regulator *R* is then either phosphorylated again with a rate *d*_*R*_ (R.VII) or binds to the specific porin of the second nutrient *N*_2_ with a rate constant of *k*_*b*_ (reaction R.V) to form the inactive compound *B*_2_; thus *R* inactivates *P*_2_. The porin-regulator compound *B*_2_ dissociates with a rate of *k*_*ub*_ (R.VI). Note that the phosphorylated version *R*^*p*^ is not explicitly represented in the model and assumed to be available in constant concentration.

The model assumes an exponential growth dynamics, whereby the rates of nutrient uptake, porin production and growth are proportional to the available biomass. This entails that biomass is conceptually best interpreted as biomass across a population rather than the mass of an individual cell. As such, it denotes a measure of the population size. The fact that nutrient uptake and porin production are also proportional to biomass implies that the volume of individual cells is considered constant. This simplifying assumption was made for reasons of model parsimony.

It can be shown (see Appendix A: Modelling the repression: Bistability) that under certain circumstances, the regulation of *N*_2_ is bi-stable and the uptake/metabolism of *N*_2_ is on or off depending on the uptake rate of *N*_1_. In this regime the system can realise sequential uptake of the two nutrient sources. At the same time, there are parametrisations of the network that lead to simultaneous uptake of nutrients.

Below it will turn out that the space limitation in the model will become crucial to the understanding of the model. The factor *L* which appears in reactions R.III and R.IV is the space-limit which represents the fact that the surface of cells can accommodate a finite number of porins only.

(1) $$ L={\frac{K_{L}}{P_{1} + P_{2} + K_{L}}}   $$

It represents the capacity of the cell to incorporate porins on its surface, averaged over a life-cycle and the population. The porin limitation mechanism does not represent a specific biological feed-back mechanism, but merely implements a general limitation scenario of the metabolism. Within the current model, this could have been implemented differently, with the same result. While the surface area for porins is most certainly limited in real cells it is unclear whether or not this is the dominant limitation scenario for cells, or whether there are others.

Throughout this contributions we use a system of differential equations to encode the model. We numerically integrate the system using the Maple 17 standard ODE solver.

### Understanding the parameter space of the model

**Fig. 2:**
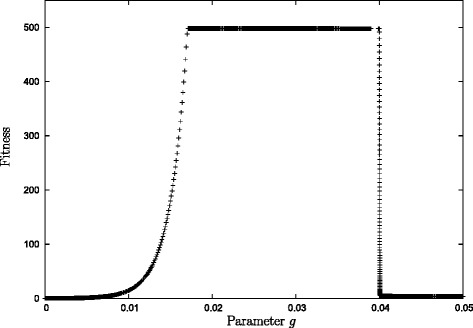
The fitness as a function of the growth rate constant *g*. To generate this graph we took a solution that evolved in the first iteration. Then we removed *N*_2_ and varied the parameter *g* leaving all other parameters at their evolved value. The model suggest that there is a phase transition between high growth and no growth

Insight into the nature of the parameter space is provided by varying the growth rate constant *g* that controls the amount of resource that is used for growth (R.VIII). Figure 2 shows the fitness of an evolved solution as a function of the growth rate for a particular solution we obtained. In this case, the fitness is high and minimally varying when *g* is within a certain intermediate range. When *g* is outside this range, either lower or higher, then the fitness is very low and again varying weakly only with the parameter. The transition between these two ranges is sudden. For other solutions we found this behaviour repeated qualitatively even though the numerical details are somewhat different.

**Fig. 3:**
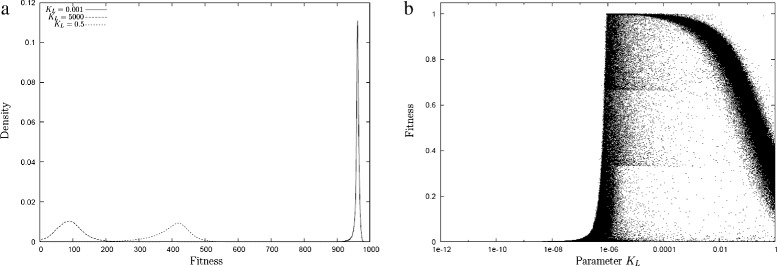
**a** The distribution of fitness of random solutions for three different values of the parameter *K*_*L*_. For small values of *K*_*L*_ corresponding to little space on the cell surface the random solutions are sharply peaked around a high fitness value converting well over 90 % of nutrient into growth. For *K*_*L*_=0.5 and *K*_*L*_=5000 random solutions peak at much lower fitness. **b** The fitness of 650000 random parameters as a function of the capacity of the uptake system. Each dot in this figure represents a simulation where parameters were chosen randomly and uniformly from the range [0,15] except for the value of *K*_*L*_ chosen randomly from the range [0,1]. The slight horizontal lines visible correspond to solutions that take up the second nutrient only (at 1/3) and the first nutrient only (at 2/3). All simulations here show solutions grown in absence of competition

We found that the porin limitation *L* (Eq. 1 and reactions R.III and R.IV) had a dominant influence on the behaviour of the system. The factor *L* takes values between zero and one and describes how close the cell is to the maximal porin limit. *L* expresses the remaining space for porins as a Hill function. The Hill parameter *K*_*L*_ is therefore the half-capacity of the cell surface for porins. We considered three different values, *K*_*L*_=0.001,0.5,5000 (see Eq. 1). The latter condition means that there is in essence no limit to the porin capacity of the cell. The first condition proved to be severely limiting and a value of *K*_*L*_=0.5 is moderately limiting. Figure 3a illustrates the impact of this parameter on the behaviour of the model. It shows a histogram of random solutions for each of the three limitation condition. For a very large value of *K*_*L*_, corresponding to practically unlimited space on the surface, random solutions have a low fitness and grow only to about 100 units of biomass corresponding to a tenth of the maximally possible. This means that they only convert one out of 10 units of nutrient into growth. This is in clear contrast to the random solutions achieved in the case of extreme limitation. In this case, the distribution peaks sharply at around 950 units, which means that these random solutions convert 95 % of the nutrients that they take-up. The moderately limited case is somewhere in-between. For very low capacities, the fitness that can be achieved is capped by the uptake time (Fig. 3b).

The effect of a low value of *K*_*L*_ is to limit the proportion of energy that can be diverted to uptake/metabolism rather than to growth. In real organisms (or more complex models) the same effect could be achieved by *any* sort of limitation of the uptake/metabolic capacity. A low *K*_*L*_ leads to slow growth and high yield, but note that slow growth is not a causal factor of the high yield. Instead, the relationship between growth rate and yield is a consequence of the dependence on the performance of the system on the energy allocation strategy (see Appendix B: Maximising flux to biomass production). In a model of a (hypothetical) organism where the energy costs are met separately from the energy costs to produce growth there would be no resource allocation problem and the total cost of uptake would not depend on the uptake speed.

Throughout this contribution the simulated cells are offered two nutrient sources which we denote by *N*_1_ and *N*_2_. We assume that *N*_1_ is of higher or equal quality as *N*_2_. Built into the model is a repression mechanism mediated by *R* (reflecting the role of dephospho EIIA ^Glc^). When *N*_1_ is taken up by the cell then *R* is produced (reaction R.I) and may bind to porins for the second nutrient (reaction R.V), preventing uptake of *N*_2_. Together with the positive feedback from the intra-cellular *N*_2_ to expression of porins for *N*_2_ this can act as an effective repression mechanism of *N*_2_ uptake in the presence of *N*_1_. This inducer exclusion only works for some parameters and requires fine-tuning in order to be effective. In our model inducer exclusion is primarily controlled by three parameters, namely: (*i*) The rate constant *k*_*b*_ with which the regulator *R* combines with *P*_2_ (reaction R.V), (*ii*) their dissociation rate constant *k*_*ub*_ (in R.VI) and (*iii*) the rate *d*_*R*_ with which *R* is phosphorylated/removed from the system (reaction VII). (The repressor *R* is produced when *N*_1_ is taken up and its production rate is therefore not a tunable parameter.)

**Fig. 4:**
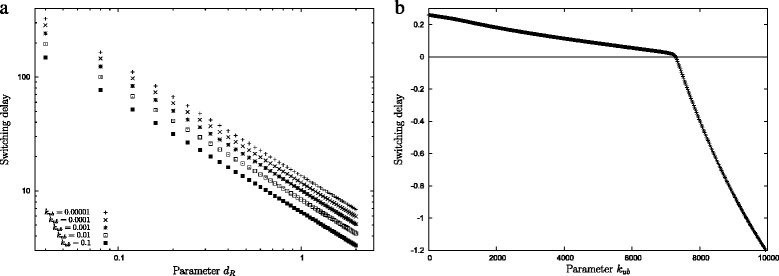
The length of the lag phase as a function of the binding rate of parameters (**a**) *d*_*R*_ and (**b**) *k*_*ub*_. The graph was obtained by using a solution that evolved regulated sequential uptake. All parameters were kept constant except for the ones indicated

The balance between these three parameters determines not only whether the system excludes the *N*_2_ inducer, but also how fast it re-activates *N*_2_ uptake/metabolism once *N*_1_ is used up—how fast it switches from *N*_1_ usage to *N*_2_ usage. Phosphorylation (controlled by the rate constant *d*_*R*_ in reaction R.VII) is the main sink of *R*. The slower this process, the more *R* there is during *N*_1_ uptake and the slower it is removed once *N*_1_ is exhausted. The uptake and metabolism of *N*_2_ cannot be kick-started as long as there is a substantial amount of *R* left. Hence, *d*_*R*_ controls the time to switch between the nutrient sources. Figure 4a illustrates this with a numerical example. The graph shows the switching time (see ‘Methods’ Eq. 2 for a definition) as a function of *d*_*R*_ for several association rate constants *k*_*ub*_. For the particular parameters considered here it suggests that the switching time is nearly inversely proportional to the removal rate constant. Significant lag-times can be achieved for very low removal rates *d*_*R*_.

The time to switch between *N*_1_ and *N*_2_ metabolism also depends on the rate *k*_*ub*_ with which the repressor and *P*_2_ dissociate. Figure 4b shows how this parameter influences the switching time. While the numerical details of the graph are specific to the particular parametrisation of the model the qualitative behaviour is generic. At low values *k*_*ub*_ determines the length of the lag-phase. Increasing the parameters beyond a particular value then leads to a situation where the switching time is negative, i.e. the two nutrients are consumed simultaneously.

### Evolving the parameters

**Fig. 5:**
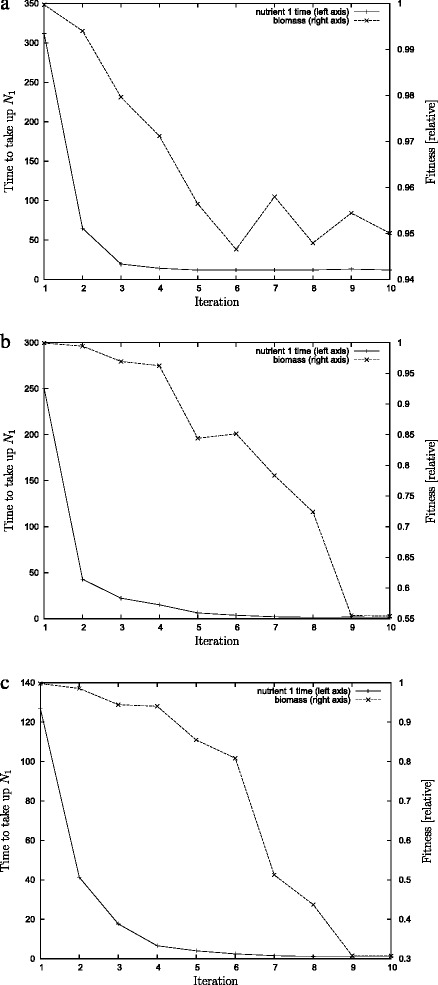
The time when nutrient 1 is used up (left axis) and the biomass produced after 500 time units (right axis). The graph shows simulations against a dummy incumbent. The time to take up nutrient 1 falls quickly during the first few iterations and then approaches a limit. **a** In the case of extremely limited space total growth falls marginally only to about 95 %, whereas there are more significant drops in the case (**b**) of moderate limitation where the efficieny falls to nearly 55 % and (**c**) in the case of no limitation where the efficiency falls to about 35 %. These graphs report the results from individual evolutionary runs

Using artificial evolution it is possible to find parameters with fitness much larger than the typical fitness as indicated by random solutions. Even for the case of the unlimited space, where random fitness clusters at around 10 % of the achievable biomass, the genetic algorithm can find solutions that achieve a fitness of about 95 % of the maximal fitness (see Fig. 5). Fitness values that high can only be achieved when the incumbent is a low-performing solution, i.e. especially during the first iteration of the GA.

For the second and subsequent iterations the competitor has to evolve against increasingly fit incumbents. By construction of the model, the incumbent and competitor directly compete for the same nutrient source and the maximal combined biomass cannot exceed the available nutrient. A direct implication of this is that any growth of the competitor is unavailable for the incumbent. In the ideal case the competitor can evolve parameters that monopolise growth and prevent the incumbent from growing at all.

**Fig. 6:**
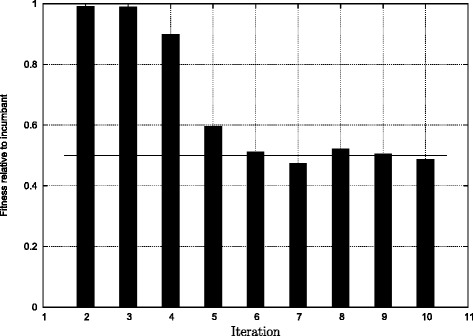
Fitness of solutions when run against their immediate predecessors. The bar over the number 2 indicates the fitness of the second solution relative to the first one calculated as *b* *m*_2_/(*b* *m*_1_+*b* *m*_2_). A value of 1 means that the incumbent (in this case solution 1) did not grow at all

To get an overview over the evolutionary dynamics of the system it is convenient to think of it as proceeding through 3 distinct phases. These phases are helpful to structure a narrative, but they are not necessarily clearly distinguishable in every instance and a more thorough account will be provided below (see Fig. 6). During Phase I competitors increase the speed of nutrient uptake and evolve to dominate the incumbents completely. They achieve this by taking up nutrient much faster than the incumbent and thus monopolise all nutrients. The incumbent achieves no or only minimal growth. After a number of iterations—typically around 4—Phase II begins. During this phase the speed of nutrient uptake has reached a system limit and it is not possible to increase the speed of uptake. In Phase II competitors no longer evolve to monopolise nutrient uptake but still outperform the incumbent in the sense that they generate more biomass. The final Phase III starts when an incumbent is so efficient that it cannot be dominated any more. In this case, competitors fail to evolve strong solutions and are sometimes even quite uncompetitive. In subsequent iterations these relatively poor solutions can then be dominated again, giving rise to stronger competitors that in turn are harder to dominate in the iteration thereafter. In this final phase there often emerges a quasi-cyclic pattern of fit incumbents followed by not so fit ones. For the purpose of this article we will predominantly concentrate on Phase I & II where the evolutionary dynamics is most interesting and relevant for real organisms. We will argue below that the third phase is an artefact of the model design and has no relevant counterpart in real organisms.

For our experiments we considered three different cell surface capacities, namely high, intermediate and low corresponding to the parameter values *K*_*L*_=5000,0.5,0.001 respectively. All parameters displayed the three phases described above. To understand the evolved solutions we tested them in the absence of competition, i.e. we ran them against a dummy incumbent that is unable to take up any nutrient. Figure 5 illustrates the typical results obtained over three iterations. It shows the time required to use up *N*_1_ for the best solution evolved during each iteration. For all three limitation conditions the time required to take up *N*_1_ drops sharply until a system limit is found.

The minimum time required to take up nutrient depends directly on the number of porins through which the nutrient is taken up and hence is determined by the parameter *K*_*L*_ (that limits the maximal number of porins expressed). To determine this minimum time we considered for each limitation scenarios six independently evolved solutions from the eighth iteration. Over each of these six different evolved solutions for each limitation we determined the average time required to be 13.04±2.34 for the extremely limited case of *K*_*L*_=0.001. For the moderately limited case the time was 1.438±0.06 and in the case of no limit we found a minimum time of 1.36±0.2. This indicates an increase of the uptake rate by a factor of ten from the extremely limited capacity to unlimited capacity.

The increase in speed coincides with a decrease in yield, i.e. fitness. Using the same amount of nutrient, competitors converted less of it into biomass. Over time, the solutions therefore evolved a more inefficient use of nutrient. In the case of extremely limited space for porins the resulting inefficiency is modest and the late iteration solutions are merely 5 % worse than the best solution. However, as the limitation on the number of porins on the cell surface is released, the efficiency losses are much higher. When there is no limitation on the number of porins, then eventually solutions utilise only 35 % of the available nutrient for growth (see Fig. 5). However in all cases, the most inefficient solutions still have a higher yield than the typical random solution. Note that inefficient does not mean uncompetitive. Those inefficient solutions outperform the more efficient solutions that evolved during earlier iterations.

### Evolution of sequential nutrient uptake

The strategy of increasing the uptake speed to outperform the incumbent finds a limit. The topology of the gene network in this model allows cells to evolve sequential uptake of nutrients by suppression of *N*_2_ uptake via *R*. Diauxic growth, if it evolves, would require that the simulated cells first take-up the preferred *N*_1_ and only then switch on take-up of *N*_2_. This strategy only makes sense when the capacity of the uptake system is limited, for example, when the number of porins on the surface is limited. Based on this reasoning one would therefore not expect to see sequential nutrient uptake to evolve in the case of the unlimited porin capacity (i.e. *K*_*L*_=5000).

A closer examination of our results shows that the case is somewhat more subtle than expected. Sequential uptake may evolve in all cases. However, unless the limitation of uptake is strong this sequential uptake is not regulated but merely a consequence of differing uptake speeds for the two nutrients. In order to gain insight into this we consider again the results of six evolutionary runs each consisting of 10 iterations. We do this for each limitation condition and count the number of competitors that show a positive switching delay when calculated according to Eq. 2; see Table 2. The table, shows a trend for a higher probability of sequential uptake evolving in the case of extreme limitation than in the case of moderate limitation. Simulations with unlimited capcitity have the lowest incidence of simultaneous uptake. Yet, unexpectedly, there are still some occurrences of unlimited uptake even in this latter case (during iterations 3–7). Hence, sequential uptake evolved in all limitation scenarios to some extent.

**Table 2:** For every limitation condition the sign of the switching time was calculated. The table shows the average over six repetitions of the evolution. A value of −1 means that all repetitions had a negative switching time. Similarly, a value of +1 means that in all repetitions there was sequential uptake of nutrients

Iteration	Extreme	Moderate	Unlimited
1	-0.67	-0.67	-1.00
2	-0.67	-0.33	-1.00
3	-0.67	**0.33**	**0.00**
4	**0.67**	**0.33**	**0.33**
5	**1.00**	-0.67	-0.33
6	**0.33**	**0.00**	-0.33
7	**0.00**	**0.33**	**0.00**
8	**0.67**	**0.33**	-1.00
9	**0.67**	-0.33	-1.00
10	**0.67**	-1.00	-1.00

Bold numbers indicate a predominantly positive delay

To check that this is not due to a bias in the evolution algorithm, we compared the results with a control where *N*_1_ and *N*_2_ have the same growth value. In this case nearly all solutions evolve simultaneous uptake of both nutrients (data not shown) irrespective of the limitation scenario.

Considering the switching time alone suggests that there is a relatively minor difference between the limited and unlimited scenario. However, a closer inspection of the results reveals that the nature of the sequential uptake in the extremely limited and in the other two cases is fundamentally different. Sequential uptake could be realised in two different ways: Firstly, either *N*_1_ is simply taken up faster than *N*_2_. If the difference in take-up speed is large enough, then it could be the case that by the time *N*_1_ take-up is finished take up of *N*_2_ has not properly started yet. Or, secondly, another way to realise sequential uptake is repression. In this case the two uptake systems do not need to differ in speed but interact via inducer exclusion, i.e. *N*_1_ uptake regulates down *N*_2_ uptake.

For as long as the nutrients on offer are fixed, the two cases may not be immediately distinguishable. Yet, upon changing conditions these would have very different properties. For example, in environments with *N*_1_≫*N*_2_ the regulated cell would still take up all of the *N*_1_ first and only then take up the other nutrient. In the unregulated case, uptake of the two nutrients would eventually be simultaneous once the amount of *N*_1_ is large enough.

**Fig. 7:**
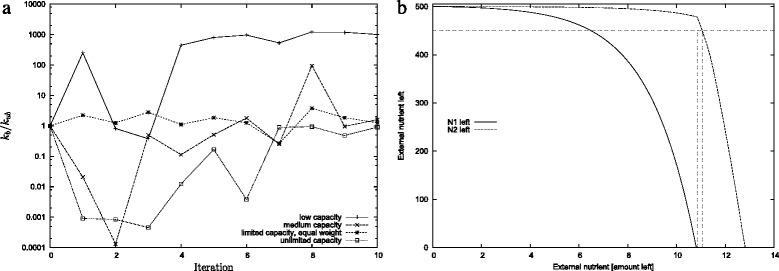
**a** The ratio *k*_*b*_/*k*_*ub*_ averaged over six different evolutionary runs. The parameter *k*_*b*_ determines how strongly repressors bind to the porins for nutrient; similarly *k*_*ub*_ determines how strongly the repressor-porin complex dissociates. Hence, the ratio is an indicator of how strongly the system represses uptake of *N*_2_ in the presence of external *N*_1_. A low value would indicate a weaker regulation than a high value. Note the logscale on the vertical axis. **b** The remaining external nutrient *N*_1_ and *N*_2_ as a function of time using an example parameter set where sequential uptake has evolved. The horizontal line indicates the value 450. The vertical line indicates the point where *N*_1_ is exhausted and where *N*_2_ crosses 450. The time difference between the two horizontal lines is the switching delay (i.e. Eq. 2)

It can also be seen directly from the parameters whether or not a solution has regulated sequential uptake or not. The crucial indicator is the ratio *κ*=*k*_*b*_/*k*_*ub*_ of the association and dissociation rate of the repressor *R* and the porin *P*_2_. Values *κ*≫1 indicate regulation. Thus it is possible to distinguish between genuine regulation and unregulated sequential nutrient uptake. We found that in the extremely limited scenario the average *κ* is significantly larger than in the other scenarios (see Fig. 7a). Indeed, we could only find a single case of regulated sequential uptake for the moderately limited scenario and no case for the unlimited case.

**Fig. 8:**
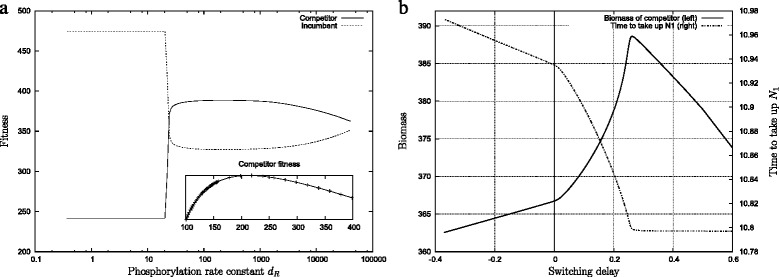
**a** There is an optimal length for the lag-phase. Along the horizontal axis we vary the parameter *d*_*R*_ that determines how fast the regulator is phosphorylated. The dependence of the fitness on this parameter shows two phases. For low values, corresponding to long lag-phases the fitness does not depend on the parameter. For higher values, there is an optimum *d*_*R*_ for the competitor. The inset shows a detailed view of the optimum. In this particular example, the parameter *d*_*R*_ evolved close to the otimal value. **b** The same data but time to take up *N*_1_ and the fitness plotted as a function of the switching delay. All data from in these plots was obtained by taking a single evolved solution and varying the parameter *d*_*R*_. All simulations here show an evolved solution and the incumbent against which this solution evolved

We have now established that regulated sequential uptake can only be realised when the parameters *k*_*b*_,*k*_*ub*_ and *d*_*R*_ are in the right relationship. Yet, there are many ways to set these parameters so that they are compatible with regulation. The question is now whether or not some of these parameter configurations are better than others. To understand this we plotted the fitness as a function of the phosphorylation rate constant *d*_*R*_ in Fig. 8a using a solution that had evolved to regulated sequential uptake. The figure indicates two different regimes. Firstly, for small values of *d*_*R*_ (on the left side of the graph) the fitness of the competitor is very low but the fitness of the incumbent is high. Within this regime both are unaffected by further lowering the phosphorylation rate. This regime can be understood as follows: The lower the value of *d*_*R*_ the longer it takes to switch on *N*_2_ uptake and metabolism, as shown above in Fig. 4a. The incumbent competes for the same *N*_2_ source and does so only a bit slower than the competitor. If the time required to switch goes above a certain value, then the incumbent will be able to take up all of *N*_2_ before the competitor can do so. The fitness of the competitor and incumbent cross where this happens. A further decrease of *d*_*R*_ then remains inconsequential because switching has become irrelevant. Hence, the low competitor fitness in this regime indicates that it only takes up *N*_1_ but not *N*_2_.

As *d*_*R*_ increases above this point, there is a change of regime. The fitness of the competitor increases suddenly with a corresponding drop of the incumbent fitness. The fitness of both incumbent and competitor become sensitive to the value of the parameter. This transition coincides with a critical value of the phosphorylation rate that allows a sufficiently fast switch to enable the competitor to take up *N*_2_ before the incumbent does. A further increase of the phosphorylation rate leads to yet another regime where the fitness is varying slowly. In this regime, the sequential uptake strategy enables the competitor to speed up uptake of *N*_1_ and get a higher share for itself. There is an optimal value *d*_*R*_ that globally maximises the fitness of the competitor (see Fig. 8a (inset)).

A complementary view can be gained by plotting the fitness not against *d*_*R*_ directly, but instead against the switching delay (see Fig. 8b). (The switching delay is not an independent variable of the system, but a consequence of a particular parametrisation and calculated by Eq. 2). Figure 8b shows that there is an optimal, non-zero delay time. In stochastic models one would expect an optimum as a result of the antagonistic relationship between the switching speed and the cost of switching. In the deterministic model we used here it is not immediately clear why there is an optimum as well.

Instead, one would expect that faster switching (i.e. shorter delay) is always better because it leads to a reduced loss of time while switching between the nutrient sources. Yet, note that the limiting case of very fast switching is simply simultaneous nutrient uptake, which is the same as no switching. A very long delay, on the other hand, is detrimental as well because it leaves all of the *N*_2_ to the incumbent.

To shed some light on this it is useful to consider how the biomass and the time to take up all of *N*_1_ change as a function of the switching delay (Fig. 8b). Below a critical switching delay this time correlates negatively with the delay, but becomes independent of it above this threshold value. This can be understood in concrete biological terms: A shorter delay entails that more *N*_2_-specific porin is on the surface during *N*_1_ uptake. This reduces the total capacity available for *N*_1_ and consequently increases the time required to take-up a given amount of *N*_1_. The point at which *N*_1_ becomes independent of the delay time is exactly where *N*_2_ uptake is effectively switched off until all of *N*_1_ is exhausted. This critical point coincides with the optimal value for the delay.

## Discussion

When cells compete with one another for limited nutrient then this leads to a competitive pressure for fast uptake and inefficient nutrient conversion. This is the case in our model where two nutrients are on offer, but would not be different in a model with only one nutrient. Less efficient and giving less yield does not mean that these later solutions are less successful evolutionarily. In a competitive situation preventing others from utilising nutrient is as important as converting nutrient into growth. Cells that take up nutrient efficiently but slowly will find themselves out-competed and irrelevant as competitors. Speeding up the usage of resources is therefore the primary adaptive effect here, whether or not there is only one or several nutrients in the environment.

Sequential take-up of nutrient is a secondary effect only. It may evolve when there are several nutrients in the environment and one is better than the other. In our simulations we saw two modes of sequential uptake. Often the more efficient nutrient is simply taken up faster than the less efficient one. Less frequently proper regulation evolved, i.e. where inducer exclusion prevents *N*_2_ uptake. A necessary condition for this to happen is severe capacity limitation of the uptake/metabolism.

### Abstraction versus realism

The question now is to what extent this emerging picture of the evolution of diauxic growth is relevant for the understanding of real systems. After all, the models we used here relied on a specific network topology, a specific abstraction of the cell model and a specific way to implement evolution. Each one of these components carries key simplifications that could materially affect the outcome of the model,

One of the central parameters we identified was *K*_*L*_ the capacity of porins. *Prima facie* it maps onto a specific part of the dynamics of cells, namely the available space for porins on the cell surface. Yet, it is not necessary to interpret its meaning relative to real systems so narrowly. The relevant dynamical effect of *K*_*L*_ is that it limits the speed of uptake. Any mechanism that limits the speed of nutrient uptake would have the same evolutionary effect. Cells certainly are subject to limits on the rate of nutrient uptake. What precisely these limits are is harder to say, but not important either, at least not for our purpose here. The role of *K*_*L*_ should be seen as representing this unknown limitation whatever its origin.

A further simplification made here is that the direct cost of regulation is ignored. One could argue that regulating cells are at a disadvantage relative to non-regulating cells because they have to carry the additional burden of the regulatory mechanism. In the present case, this would be the cost of the phosphorylation-dephosphorylation cycle. This cost was not included in the present model because it is not clear how to define it rigorously. Moreover, it is also unclear what proportion of any cost is attributable to sequential uptake regulation, rather than to some other function of the PTS. That said, in a first approximation, one can think of the regulation cost of as reducing the growth value of glucose (i.e. *N*_1_ in our model) relative to the second nutrient because an important factor of this cost is the phosphorylation/dephosphorylation cycle.

Connected to this is the representation of growth and biomass. In real cells, growth is the result of a myriad of biochemical reactions, metabolic pathways and regulatory interactions. In our model all this is compressed into a single reaction (R. VIII). Such idealisations are not appropriate when the aim of the model is to elucidate the detailed biochemistry of a particular organisms. However, here we are interested in general strategies for a large class of organisms. Very detailed biochemical models would be inappropriate because there is a large number of ways to implement one and the same strategy. Biochemical detail is therefore not only irrelevant but also hindering insight by hiding the essential behind a deluge of accidental features.

The purpose of the present contribution is precisely to understand general strategies and not details of their biochemical implementation. For example, we predict that cells will evolve to take up nutrients faster when under competitive pressure. In order for this prediction to be relevant, all we need to assume is that real metabolic networks can be tuned to varying nutrient uptake speeds. This is a weak assumption in that metabolic and other networks primarily rely on catalysed reactions whose rates depend on the concentration of some protein. The production of those can be scaled up and down within some limits thus adjusting the speed of the network. It should be noted, however, that a spatially structured population may escape this race to faster uptake (see [22]). It would be interesting to model whether or not such spatially structured populations still lead to the evolution of diauxic growth.

Another question relates to GAs as a model of evolution. A key feature of GAs is that they rely on an explicitly defined fitness function. Real systems do not evolve according to a fitness function but they will be subject to many simultaneous adaptive pressures that emerge in the environment in which they live. Indeed, it is chronically difficult to understand the adaptive pressures that shape the evolution of real organisms. One cannot therefore have much confidence that any fitness function captures the adaptive pressures that real cells face. However, for the purpose of this contribution, the weakness of GAs as models of evolution turns out to be their strength: The single fitness function allows the modeller to specify a well defined fitness criterion and thus to investigate a very particular hypothesis about the effect of adaptive pressures on the evolution of diauxic growth.

Another problem of GAs with regards to the current application is that they do not lend themselves to model the evolution of a heterogeneous population. Our approach with iterative rounds of evolutions circumvents this problem to some extent, but at the price of introducing some artefacts. This concerns specifically Phase III of the evolution where fitness starts to oscillate over iterations. In a heterogeneous population such oscillations cannot occur. Depending on the mutation rate suboptimal solutions still emerge, but they would manifest themselves as a part of the Eigen-Schuster quasi-species [23].

### Evolution towards inefficiency

The competitive evolutionary dynamics forces cells away from slow (but efficient) growth to rapid (but wasteful) growth. This means that optimal in evolutionary terms is not necessarily maximally efficient. The question is now whether or not real cells have evolved similar inefficiencies. One could argue that bacterial lab-strains are isogenic and do not experience any competition. This is not so. Given the high mutation rates in bacteria, even in populations of lab-strains there will be a constant adaptive pressure from mutants arising within the population, particularly if the strain has been grown frequently in exponential growth.

This also leads to an immediately testable prediction from our model: Wild-type cells are not optimised for yield, but for growth rate and it is possible to obtain cells with increased cell yield from artificial evolution experiments designed to avoid (or minimise) cell-cell competition. The problem is that designing an experiment to test this is difficult. One possibility is to grow cells in a chemostat at low cell concentrations and to implement a population-based selection mechanism. Altogether, it is perhaps easier to increase competition rather than to avoid it. Indeed, there is empirical evidence (for yeast) that populations shift to a lower yield when in a competitive environment [24].

The question of metabolic efficiency of cellular metabolism has recently attracted significant interest in a different context. It has long been known that cells sometimes switch to apparently less efficient metabolic pathways (for example Molenaar *et al.* [25] or Gottstein *et al.* [26]) suggesting metabolic inefficiencies. Upon closer inspection it was then found (at least in some cases) that these apparently less efficient pathways are indeed optimal [26] once the costs of protein production are taken into account.

*Prima facie* this appears to be similar to what we observed. In reality, the apparent inefficiencies discussed in [25, 26] are in no clear relationship to the evolved inefficiency discussed here. Their argument is about mechanisms of converting nutrient into usable energy in the cell and relies on detailed accounting across several biochemically feasible pathways. The metabolic pathways in our model are too coarse grained to represent this. For this reason alone the evolution of inefficiency we observe must be a different effect. Furthermore, our models predict a true inefficiency that cannot be resolved by more detailed accounting.

### Evolution of the lag-phase

In our evolutionary experiments we never observe the emergence of a substantial lag-phase, not even in those instances where cells evolve sequential nutrient uptake. (Upon closer inspection of the model output it can be seen that the growth rate drops somewhat during the nutrient switch (data not shown), but this happens only for a very brief moment and is a very minor effect only.) This suggests that lag-phases are not a generic phenomenon of deterministic models, but require additional assumptions.

One possibility is that the lag-phase is simply a manifestation of delays inherent to gene expression. In our simulations these delays are not modelled. While possibly a contributing factor, delays are unlikely to be an exhaustive explanation because the length of the lag-phase appears to be, at least partially, under evolutionary control, as discussed in the ‘Background’ section.

Another lead is given by recent experimental evidence [4] suggesting that at the level of the individual cell a clearly discernible lag-phase does not exist. Instead, one can observe a wide distribution of switching times ranging from 0 to very long. This links the lag-phase to stochastic effects due to noise in gene expression [27–30]. In the presence of noise fast regulation requires energy input [31] leading to a trade-off between speed and cost of a biological computation [13, 32]. This suggests an explanation for the population-level lag-phase that is rooted in stochastic models of gene expression.

## Conclusion

Given our model there are three requirements for regulated sequential uptake to evolve. Firstly, there must be competition. Secondly, uptake/metabolism need to be capacity limited. Thirdly, there must be a quality difference between the nutrients. Yet, even with those conditions all fulfilled, regulated uptake will only evolve sometimes, not always. Moreover, even without capacity limitations unregulated sequential uptake may evolve caused by large differences in the uptake speed of the two nutrients.

Moreover, we could establish that in competitive growth situations as modelled here there will be a drive to increase the growth rate at the expense of yield. Sequential uptake of nutrient is only a secondary effect that evolves once the potential for speed increase has been exhausted. Even if sequential nutrient uptake evolves, substantial lag-phases are sub-optimal in our set-up and we conjecture that a lag-phase is a stochastic phenomenon and a consequence of the inherent computational cost of such stochastic regulation systems.

## Methods

### Artificial evolution

Artificial evolution both in wet-lab experiments [14, 17, 33] and *in silico* [34–38] has been found useful to explore the fitness landscape of organisms. In simulated evolution investigators usually evolve both the structure of the network (i.e. the network topology) and its parametrisation (i.e. the numerical details). This is useful, for example, when one is interested in finding a biochemical reaction system that performs a particular task (i.e. a biochemical oscillator) and one is not concerned about modelling a particular mechanism. In contrast, here we are interested in a particular strategy and we ask a very specific question: “How are cellular resources allocated to uptake/metabolism and to growth?” In order to explore the space of possibilities, it is not necessary to evolve networks of different topology. In fact, this would inflate the search space, increase computational costs and complicate the analysis, without much corresponding extra insight to counter-balance. Hence, here we limit ourselves to a minimal but biologically plausible biochemical network that allows for the strategy in question to evolve and also allows us to explore allocation strategies.

We evolve parameters for a solution by using a genetic algorithm (GA) [39]. GAs are a family of nature inspired heuristic optimisation algorithms, and are a common method in computer science to solve practical problems. While they are loosely based on the idea of natural evolution, they are not a good model of how natural evolution proceeds. However, for our purpose they are the ideal tool because they make it possible to impose a specific adaptive pressure on the evolving entities, and hence to address a very particular hypothesis. As such, they enable us to explore precisely under which conditions diauxic growth evolves.

GAs operate on a population of candidate solutions. Each solution is assigned a *fitness* according to a user-determined fitness function. A new population is obtained from the existing one by choosing candidates based on their fitness values and subjecting them to *mutations* and *crossover* with a given probability. In the present case, a mutation is a small adjustment of the parameter value (subtracting or adding a random number of up to 10 % of the parameter value); if the mutation results in a parameter value >15 or <0 then the respective parameter is set to 15 or 0. Crossover produces a new offspring solution from two randomly chosen parents. This is done by creating a new set of parameters from a sub-set of parameters from parent 1 and the complementary sub-set from the other parent. Thus produced offspring may then also be subject to point mutations.

During the selection step an unmodified and a mutated version of the highest fitness solution is always retained for the next generation. The selection algorithm proceeds by choosing a random parent cell from the existing population. The chosen solution will be removed from the original population. Next, with a probability *P* the solution will be swapped for a randomly generated solution and placed into the new population. Here we choose *P*=1−*f*/*f*_*m*_ where *f* and *f*_*m*_ are the fitness of the solution in question and the maximal fitness in the population. If the chosen solution is not swapped then with a probability of 0.5 a second solution is chosen randomly and crossover is performed. The newly formed offspring is then subject to mutation with a probability of 0.2. Alternatively, if the solution is not chosen to be the parent of a new crossed-over solution it is mutated with a probability of 0.2 and then added to the new population. Once the new population has reached the determined size then each solution is assigned a fitness value and selection starts over. In the simulations reported here, the algorithm is run for 5000 generations, i.e. 5000 new populations. The population size was chosen to be 50. As in all genetic algorithms the initial population was assigned random parameter values uniformly drawn from the interval [0,15]. While we kept these parameters in all simulations reported here, the performance of the GA is not sensitive to a variation of these parameters and will perform well for a wide range of mutation/crossover probabilities.

The fitness function chosen here was the amount of biomass after 500 time units when run competitively against a fixed solution. The above system of equations specified one cell that draws nutrient from the environment. In our simulation the competitor was implemented by adding another set of equations as above that works independently but consumes the same external nutrients *N*_1_ and *N*_2_. Hence, the two solutions compete with one another for these external nutrients. Only the parameters of one of these two solutions (henceforth referred to as the *competitor*) were evolved, whereas the other solution (the *incumbent*) was kept fixed throughout the evolutionary run.

**Fig. 9:**
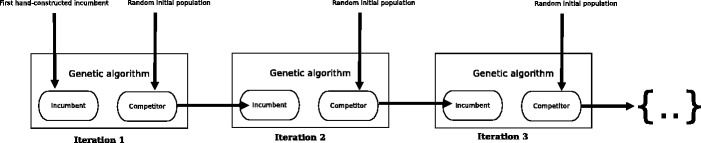
Schematic representation of the competitive genetic algorithm used here. The incumbent is the fittest competitor from the previous run of the GA

We performed each evolutionary simulation in 10 iterations. Each iteration consists of running a genetic algorithm for 5000 generation. Once finished, we located the best solution (i.e. the competitor that produced the highest biomass). This best solution was then used as the incumbent in the following iteration (see Fig. 9). The incumbent in the first iteration was a hand-constructed unfit solution that was designed to be unable to take up any nutrient from the environment. Hence, the competitor during the first iteration did not face any competition.

All parameters were allowed to take any value from the interval [0,15]. The parameters *k*_*b*_,*k*_*ub*_,*d*_*R*_ were multiplied by 2000. This reflects the fact that the relevant reactions happen on a time-scale much faster than gene expression. While most parameters were evolvable, we kept some fixed. Unless indicated otherwise, these have the same value in all simulations reported here. The parameters are: The maximal uptake rate of a porin $k_{N_{i}}$ (set to 15 throughout), the Hill exponent of all the Hill functions *H* (set to 2), total capacity of the cell surface for porins (parameter *K*_*L*_). For the latter we considered three values, namely 5000,0.5 and 0.001, corresponding to unlimited, moderately limited and extremely limited capacity. The total amount of obtainable energy was kept fixed throughout at 1000 during the evolutionary runs. However, at the beginning of each generation, the amount of *N*_1_ was randomly chosen from the range [100,900]. The amount of *N*_2_ was then chosen so as to yield the energy equivalent of *N*_1_=1000. We assumed *N*_2_ to have half the energy of *N*_1_. Hence, if *N*_1_ was set to 500 then *N*_2_ was chosen to be 1000.

To analyse the properties of solutions that evolved the fittest individual from each iteration was chosen and then run with both *N*_1_ and *N*_2_ set to 500 to determine the following properties: The biomass when grown in isolation; the biomass when grown against its competitor (i.e. the solution against which it evolved); the time $T^{(1)}_{\textrm {\tiny off}}$ when *N*_1_ reached a value of 1; the time $T^{(2)}_{\textrm {\tiny on}}$ when uptake of *N*_2_ started. In order to allow for some “leak uptake,” we defined the start of *N*_2_ when it reached a value of 450. Based on this, we defined the *switching time* as follows:

(2) $$ T= T^{(2)}_{\textrm{\tiny on}}-T^{(1)}_{\textrm{\tiny off}}   $$

The switching delay time is illustrated in Fig. 7b. Henceforth, we will consider uptake to be “sequential,” when the switching delay is positive, and “concurrent” otherwise. The precise value of 450 is arbitrary to some extent. Disallowing any leak rate (i.e. choosing the threshold to be 499) would be not very informative. It would indicate that *N*_2_ was switched on when in fact only a little bit was taken up. On the other hand, too generous a value (e.g. a threshold of 100) would misjudge the time when *N*_2_ started to be taken up. As will become clear in “Results” the conclusions drawn from the simulations will not depend on the precise threshold value assumed.The current value was chosen as a reasonable compromise based on many observations of full model results.

## Appendix A: Modelling the repression: Bistability

Uptake and metabolism of *N*_2_ is regulated by *R* associating with *P*_2_ thus making the porin unavailable. In a simplified model of this one can show that expression of *P*_2_ is bi-stable with two stable states corresponding to *P*_2_ being expressed or not being expressed. The switch between between the steady states depends on the association rate of *R* and *P*_2_, We assume that an external nutrient *N* is taken up with a rate of *E*·*l* where *E* is the number of specific porins for this nutrient and *l* is some rate constant; here we choose the units such that the fixed concentration of the external nutrient is 1. We denote this internalised nutrient as *P*. Both *P* and *E* are broken down with a specific rate *d*_*X*_. This breakdown of *E* can be interpreted as modelling the binding with the (not represented) regulator *R* in the limiting case of a vanishing dissociation rate.

(3) $$\begin{array}{@{}rcl@{}} \dot E&=& k_{1} {\frac {P^{h}}{P^{h} + K^{h}}} - d_{E} E \end{array} $$

(4) $$\begin{array}{@{}rcl@{}} \dot P &=& El - d_{P} P \end{array} $$

Within the area of feasible parameters, the system can display bistable behaviour when the Hill exponent *h*>1. In this case, there are three steady state solutions, two of which are stable. In the case of *h*=2 the system has the following steady state solutions.

$$P^{*}_{\pm}={\frac {l\pm \sqrt {-4\,{d_{P}}^{2}{d_{E}}^{2}{K}^{2}+{l}^{2}}}{2d_{P} d_{E}}} $$

These are real as long as the incoming flux of nutrients is large enough, i.e. *l*>2*d*_*E*_*d*_*P*_*K*. Beyond that, no solution exists.

## Appendix B: Maximising flux to biomass production

Central to the evolution of the uptake system is the resource allocation problem. Here we present a simple, but analytically solvable model of resource allocation in the cell. We assume that the resource *N* is taken up by porins *P* proportional to the amount of external nutrient *N*. This kinetic approximates the Hill equations during early periods of uptake when the porins are saturated. Internal nutrient *P* is converted into biomass with a rate *k*_2_ and porins with a rate *k*_3_. This can be formulated by the following two differential equations.

(5) $$\begin{array}{@{}rcl@{}} \dot E &=&k_{1} N P - (k_{2} + k_{3}) E \\ \dot P &=& k_{3} E - k_{4} P \end{array} $$

We assume that *k*_1_=*k*_4_=1 which simplifies the analysis without affecting the result in a fundamental way. The system of equations can then be solved analytically. Assuming the initial condition *E*(0)=1,*E*(0)=0 we obtain:

(6) $$\begin{array}{@{}rcl@{}} P(t) &=& {\frac{1}{2}} \left(e^{(a-1)t} +e^{-(a+1)t} \right)\\ E(t) &=& {\frac{a}{2(k_{2} -1)}} \left(e^{(a-1)t} +e^{-(a+1)t} \right) \end{array} $$

here *a* are given by

$$a=\sqrt{N - Nk_{2}} $$

This is a real number for *k*_2_≥1. The number of porins *P*(*t*) grows exponentially in time for $k_{2} < {\frac {N-1}{N}}$. We are interested in the flux of the system towards biomass which is given by:

(7) $$ F_{\textrm{\tiny bm}} \sim k_{2} \cdot N \cdot P(t)  $$

**Fig. 10:**
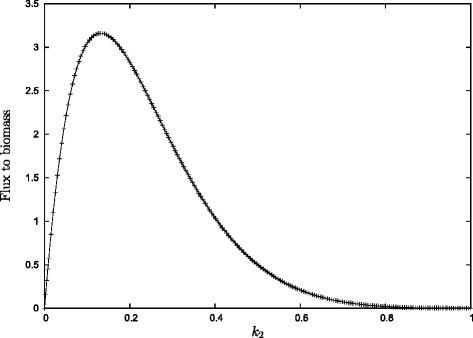
Keeping *t*=1,*n*=2 we plot the flux to biomass as a function of *k*_2_. As predicted there is a clear optimal value. This optimal values shifts to the left with time

Hence, for any *T*=*t* the flux is zero for *k*_2_=0. When *k*_2_=1 then *P*(*t*)∼ exp(−*T*). Expanding *P*(*t*) into a Taylor series around *k*_2_=1 it can be seen that for all *t*=*T* it is the case that *P*(*t*) increases for decreasing *k*_2_ in the neighbourhood of *k*_2_=1. Consequently, there is a value $0<k_{2}^{\textrm {\tiny max}}<1$ with maximum flux to biomass (Fig. 10).
